# Generation and Propagation of MALDI Ion Packets Probed by Sheet-Like Nanosecond UV Laser Light

**DOI:** 10.5702/massspectrometry.A0071

**Published:** 2018-12-26

**Authors:** Tatsuro Shirota, Kennosuke Hoshina

**Affiliations:** 1 Faculty of Pharmaceutical Sciences, Niigata University of Pharmacy and Applied Life Sciences, 265–1 Higashijima, Akiha-ku, Niigata 956–8603, Japan

**Keywords:** MALDI, proton transfer, CHCA, photodissociation, Time-of-Flight (TOF) mass spectrometry

## Abstract

A sheet-like ultraviolet (UV) probe laser is used to investigate the ejection and propagation of ion packets of matrix CHCA, which are produced by matrix-assisted laser desorption and ionization (MALDI). Laser irradiation of the expanding MALDI plume induced photodissociation of the CHCA-related ions, which existed in a sheet-like volume, leading to their absence in their MALDI signal profiles. The MALDI spectra were measured under varying conditions: the temporal delay of the lasers and the distance of the sheet-like probe laser from the MALDI sample surface. It was found that the center of the (CHCA)H^+^ packets were ejected at 46±11 ns after MALDI laser irradiation, while the (CHCA)_2_H^+^ packets were ejected at 64±12 ns, regardless of the magnitude of acceleration static high-voltage in 3.5–5.5 kV. This suggests that (CHCA)_2_H^+^ is formed by a proton transfer reaction from (CHCA)H^+^ to (CHCA)_2_ in the heated condensed phase and/or near the surface. This study represents the first experimental determination of ion ejection time in the MALDI process, which is also applicable to other species in the MALDI plume.

## INTRODUCTION

Matrix-assisted laser desorption and ionization mass spectrometry (MALDI-MS) is widely used in biological sciences as a technique for ionizing and detecting nonvolatile high-mass biomolecules, such as peptides and proteins, with minimal fragmentation.^1–3)^ After the matrix and sample mixtures are irradiated by the laser, the resulting liquid phase or dense gas plume will produce analyte ions by proton transfer from the protonated matrix molecules to the intact analyte molecules.^4–9)^ However, the time scale of matrix and analyte ion formation processes have not been elucidated in the rapidly changing conditions that are inherent in the MALDI process, which includes ultraviolet (UV) laser absorption by matrix molecules and a rapid increase in temperature, followed by the melting and vaporization of polycrystals.^10–12)^ It was demonstrated that molecular ion ejection mainly occurs from fragment clusters which are formed just after expansion of the target crystal through laser heating rather than evaporation simply from the surface.^10,13–24)^

According to a theoretical study, the interrelation between the physical and chemical processes in MALDI affects the signals and detection sensitivity.^25)^ However, the formation of protonated ions is likely to be completed within tens of nanoseconds near the laser-irradiated surface^25)^; therefore, it is difficult to trace this quick chemical process in the MALDI plume which expands only to several tens of μm from the surface.^26,27)^ Even so, the expanded MALDI plume may exhibit the memory of every chemical reactions that do not necessarily proceed on the same timescale.^9,19,28–30)^ Spatiotemporal distribution that depends on chemical species must contain information to clue the chemical processes in a hot and dense MALDI plume expanding just after laser irradiation.

A few successful investigations of the physical and chemical processes in MALDI have been conducted by probing the plume. Puretzky *et al.* measured the spatiotemporal distribution of the expanding neutral species using laser-induced fluorescence imaging 10 μs after laser irradiation and estimated their initial velocity to be 500–1000 ms^−1^.^28,29)^ Bökelmann *et al.* investigated the (DHB)H^+^ distribution 1 μs after the laser irradiation of substance P and DHB mixture and concluded that proton transfer efficiently proceeds from (DHB)H^+^ with low initial velocity to the analytes.^30)^ These previous studies have probed the propagation of MALDI plumes without an accelerating electric field in which more than several μs was required to propagate to a detectable region that resulted in a temporal resolution in the order of 1 μs. Therefore, the ion packet must be extracted and accelerated to trace the generation/ejection processes of ionic species from the surface that was observed over a period of several tens of ns with a comparable temporal resolution.

In the present study, a method that uses a sheet-like probe UV laser is proposed to trace the propagation of ion packets that are ejected by the MALDI process. Based on the spatiotemporal position of the (CHCA)H^+^ and (CHCA)_2_H^+^ ion packets, the time lag that was required to release the packets from the MALDI target surface after laser irradiation was also examined.

## EXPERIMENTAL

### Sample Preparation

α-Cyano-4-hydroxycinnamic acid (CHCA), acetonitrile with 0.1% trifluoroacetic acid (TFA), and ultrapure water that were purchased from Wako Pure Chemical Industries, Ltd. were used during the current study; CHCA was used without further purification. The MALDI samples were prepared using the dried droplet method^31)^ at room temperature under atmospheric pressure. Specifically, a 40-μL CHCA solution (50 μmol/mL in 1 : 1 solution of acetonitrile with 0.1% TFA and ultrapure water) was deposited on a stainless steel stage. The typical size of a dried sample, which contained only the matrix molecule of CHCA, exhibited a diameter of 5 mm.

### Measurement

**Figure 1:**
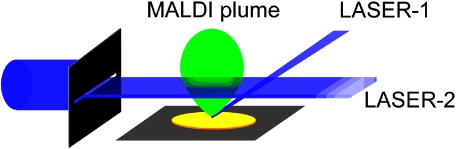
Fig. 1. Experimental scheme for probing the MALDI plume using a sheet-like UV laser.

Figure 1 shows the experimental setup for this study. The MALDI samples were irradiated with the 3rd harmonic of Q-sw Nd:YAG laser light (LASER-1, 355 nm, 5 ns, 10 Hz, Quanta-Ray GCR-3; Spectra-Physics) at an incident angle of 30°, which induced the MALDI process. The laser spot size on the sample surface was estimated to be 1 mm in diameter, which indicated a laser fluence of 7.0–7.5 mJcm^−2^ per pulse. The ions that were ejected through the MALDI process were typically accelerated at +4.5 kV (3.54×10^5^ Vm^−1^) and were detected by the microchannel plate through a linear-type time-of-flight (TOF) mass spectrometer (Jordan TOF Products, Inc.). The ion signals were further digitized (5 GS/s) on an oscilloscope, transferred to a PC, and averaged over approximately 20 shots.

Another 3rd harmonic of a YAG laser beam (LASER-2, 355 nm, 5 ns, 10 Hz, Quanta-Ray INDI; Spectra-Physics) that was cut out with a 1-mm slit, which generated sheet-shaped laser light (7 mm in length and 1 mm in height), was introduced into the MALDI plume that was generated by the MALDI laser irradiation, as shown in Fig. 1. The laser fluence at the irradiation spot was estimated to be 100–115 mJcm^−2^ per pulse. The LASER-1 and LASER-2 pulses were synchronized with each other by a delay generator (DG645, Stanford research Co.) with a temporal jitter of 1 ns. When the ions in the sheet-like volume that were irradiated by LASER-2 were dissociated into smaller fragment ions, a partial decrease in the number of the ions in the MALDI plume was expressed as a partial absence of its signal profile in the MALDI-MS spectrum. Because this information was obscured using a reflectron-type mass spectrometer, which can compensate for the spread of signal profile due to the initial velocity distribution, the linear-type spectrometer was chosen.

The MALDI-MS spectra can be measured as a function of Δ*t* and *z*, where Δ*t* is the temporal delay of LASER-2 from the time at which the temporal center of LASER-1 light pulses irradiate the sample surface and *z* is the distance of the sheet-like LASER-2 beam path with a thickness of 1 mm from the sample surface. The propagation of ion packets can be tracked by measuring (Δ*t*, *z*) when the center of the MALDI signal is missing.

## RESULTS AND DISCUSSION

### Observed MALDI-TOF spectra

**Figure 2:**
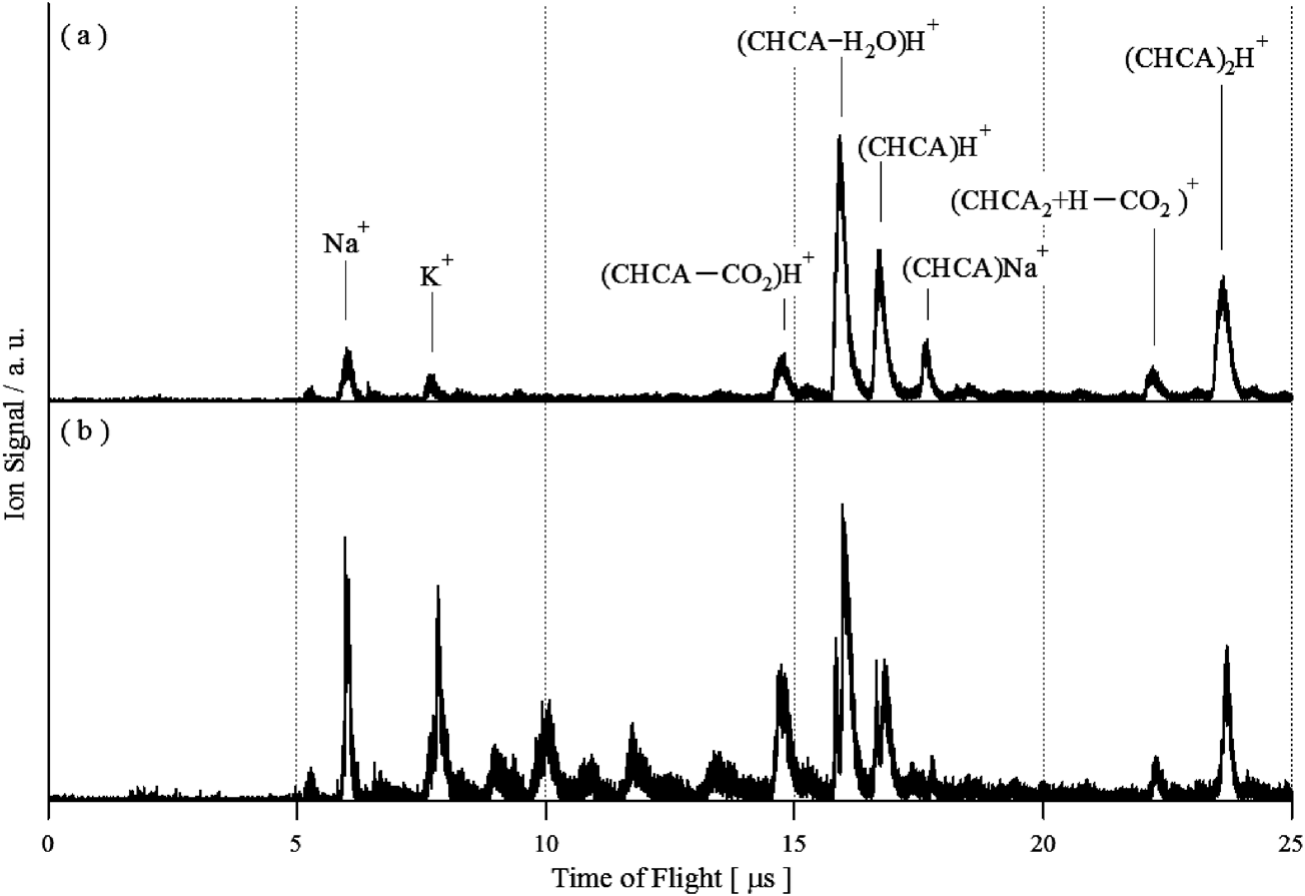
Fig. 2. (a) The MALDI-TOF spectra of CHCA without probe laser and (b) with probe laser irradiation (b) at Δ*t*=100 ns and *z*=0.6–1.6 mm.

Figure 2a shows the MALDI-TOF spectrum of CHCA obtained by LASER-1 irradiation. The typical MALDI signals of (CHCA-H_2_O)H^+^ (*m*/*z* 172), (CHCA)H^+^ (*m*/*z* 190), (CHCA)_2_H^+^ (*m*/*z* 379), and (CHCA)Na^+^ (*m*/*z* 212) are detected. It is well known that the alkali metal adduct signals originate from the impurities in samples or instruments. Because a linear-type mass spectrometer was used, each peak exhibited a wide spectral width because of the initial velocity distribution. Figure 2b shows a MALDI-TOF spectrum that is obtained when LASER-2 is irradiated after LASER-1 at *z*=0.6–1.6 mm and a temporal delay of Δ*t*=150 ns. *z*=0.6–1.6 mm indicates that the sheet-like laser with a thickness of 1 mm covers the region of *z*=0.6 to 1.6 mm. This was the closest position of LASER-2 at which measurements could be conducted without any MALDI signals from LASER-2.

There are two drastic differences in Fig. 2b relative to Fig. 2a, which include the appearance of small signals in the low-mass region and the partial absence of the CHCA-related ion peaks such as (CHCA)H^+^. The appearance of small signals by LASER-2, which were assigned to the C*_n_*H*_m_*^+^ series of fragment ions, and the partial absence in the CHCA-related peaks were observed in the same spectra throughout the present measurement. This suggests that a part of the ion packet existed in the region of *z*=0.6–1.6 mm and that it was reduced by photodissociation, leading to the absence of the (CHCA)H^+^ signals and the appearance of the fragment ion signals. Based on the extent of parts that were missing in the peaks, the volume irradiated with a thickness of 1 mm by LASER-2 was observed to be approximately one-third of the full size of the ion packet that propagated in the *z*=0.6–1.6 mm region.

### MALDI-TOF spectra measured by changing *z* and Δ*t*

**Figure 3:**
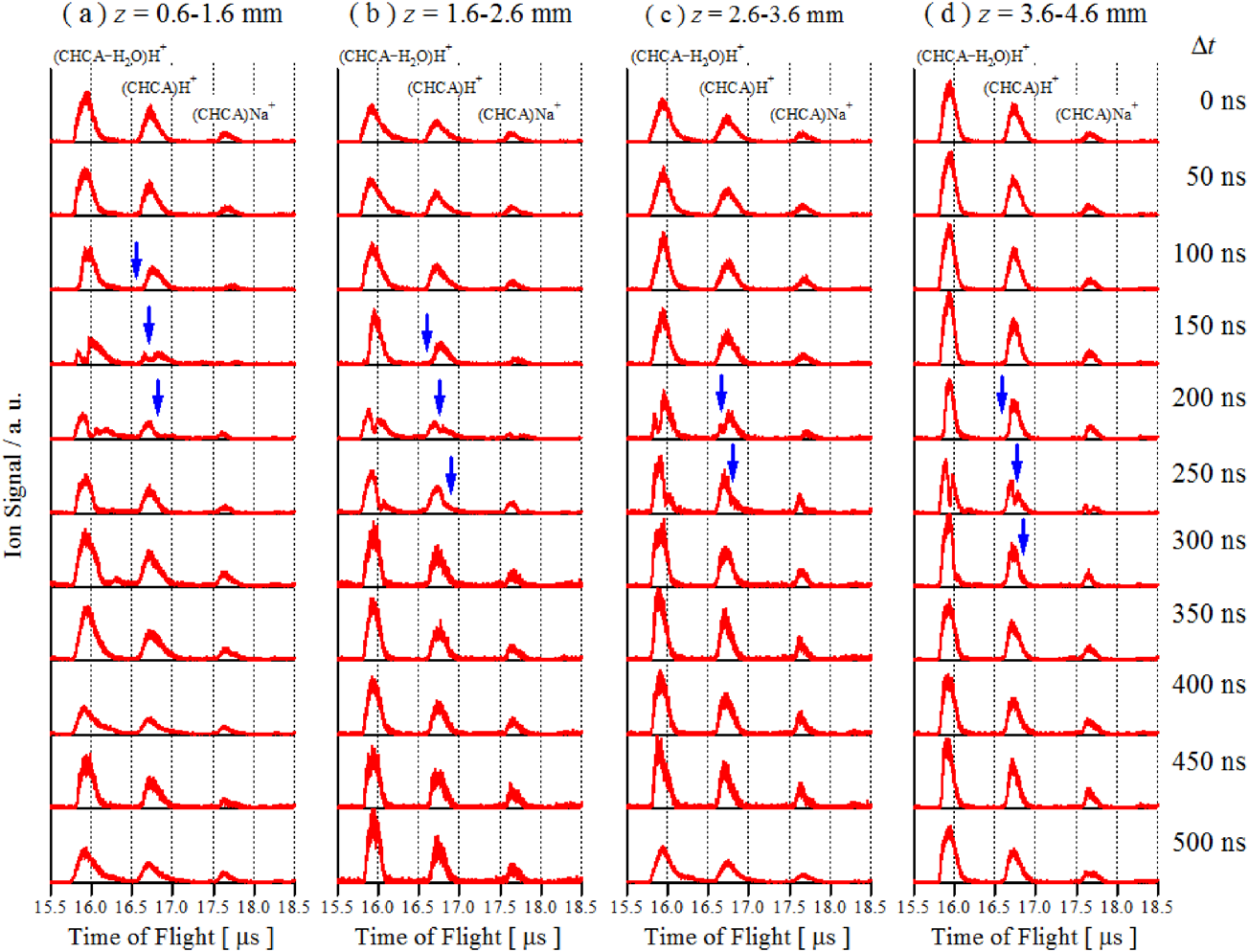
Fig. 3. The MALDI-TOF spectra in the region where (CHCA)H^+^ signals are observed with the probe laser irradiation that is measured by varying Δ*t* at a fixed *z* to (a) 0.6–1.6 mm, (b) 1.6–2.6 mm, (c) 2.6–3.6 mm, and (d) 3.6–4.6 mm.

A series of MALDI-TOF spectra were measured by varying Δ*t* from 0 to 500 ns with intervals of 50 ns at a fixed *z* value, and the comparison of the spectra is presented in Fig. 3. The arrows show the positions at which the missing ion signals are observed in (CHCA)H^+^ by irradiation using LASER-2. At *z*=0.6–1.6 mm, the missing position began to appear on the left part of (CHCA)H^+^ peak at Δ*t*=100 ns, which corresponded to the time at which the head of the ion packet was irradiated and at which the ions were dissociated by LASER-2. The missing position moves to the central part of the MALDI signal at Δ*t*=150 ns and the right part at Δ*t*=200 ns, corresponding to LASER-2 irradiation at the central and the back parts of the ion packet, respectively. This indicates that the packet passed through the region of *z*=0.6–1.6 mm within the interval of Δ*t*=100–200 ns. The temporal delay of the observed partial signal shifts to longer Δ*t* as *z* increases; Δ*t*=100–200 ns at *z*=0.6–1.6 mm, Δ*t*=150–250 ns at *z*=1.6–2.6 mm, Δ*t*=200–250 ns at *z*=2.6–3.6 mm, and Δ*t*=200–300 ns at *z*=3.6–4.6 mm. Because *z* corresponds to the propagation distance of the ion packet, it is reasonable to expect longer Δ*t* for partial signals as *z* increases. Similar partial missing signals were observed in the (CHCA-H_2_O)H^+^ and (CHCA)Na^+^ peaks. There are no new signals in the TOF spectra of (*z*, Δ*t*)=(0.6–1.6, 500) with the second laser irradiation. In addition, fragment ions seen in the lower mass region appear only when ion missing are observed in the profiles of (CHCA)H^+^, (CHCA)_2_H^+^. This indicates that post-ionization of neutral species does not occur in the present observation.

**Figure 4:**
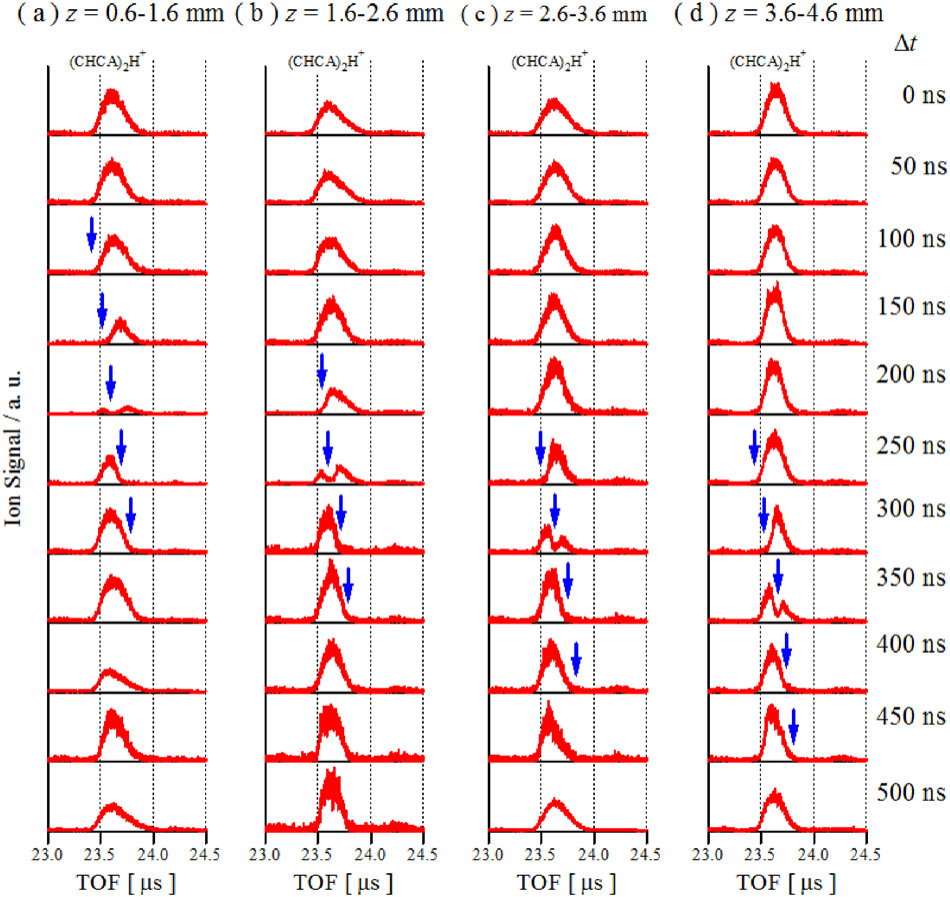
Fig. 4. The MALDI-TOF spectra in the region where (CHCA)_2_H^+^ signals are observed with the probe laser irradiation that is measured by changing Δ*t* at a fixed *z* to (a) 0.6–1.6 mm, (b) 1.6–2.6 mm, (c) 2.6–3.6 mm, and (d) 3.6–4.6 mm.

Figure 4 shows the TOF region of (CHCA)_2_H^+^. In addition, the missing signals can be labeled using arrows. The partial missing signals were observed at Δ*t*=100–300 ns when *z*=0.6–1.6 mm, Δ*t*=200–350 ns when *z*=1.6–2.6 mm, Δ*t*=250–400 ns when *z*=2.6–3.6 mm, and Δ*t*=250–450 ns when *z*=3.6–4.6 mm. The partial missing signal of (CHCA)_2_H^+^ exhibited a longer Δ*t* than that exhibited by (CHCA)H^+^, indicating that the propagation velocity of the former was smaller. It is because of its higher mass, which was approximately double that of the latter.

### Propagation of ion packets

**Figure 5:**
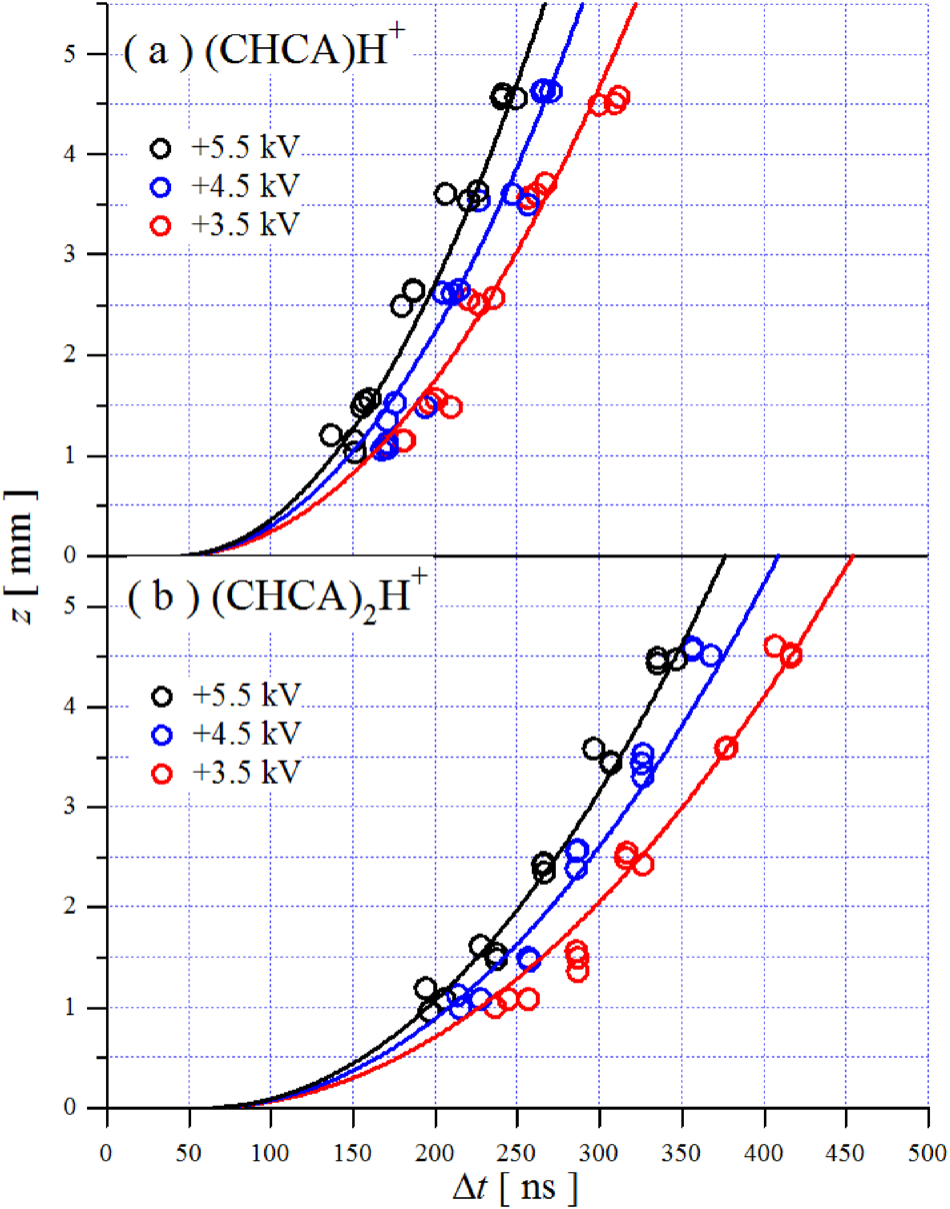
Fig. 5. Plots of (Δ*t*, *z*) at which missing signals were observed in the center of the MALDI profiles for (a) (CHCA)H^+^ and (b) (CHCA)_2_H^+^ at three acceleration voltages, +3.5, +4.5, and +5.5 kV. The solid lines are the simulations of ion propagation when *t*_E_ is set to be 45 ns for (CHCA)H^+^ and 65 ns for (CHCA)_2_H^+^.

The missing of the profile center at (*z*, Δ*t*) indicates that the center of the ion packet passes position *z* at time Δ*t*. Therefore, we surveyed the condition (Δ*t*, *z*) at which the center of the (CHCA)H^+^ profile was observed to decrease by changing *z* at a 1-mm step. Figures 5(a) and (b) plot (Δ*t*, *z*) for (CHCA)H^+^ and (CHCA)_2_H^+^, corresponding to the propagated distance of the ion packet center, which was obtained for the three acceleration voltages of +3.5 kV (2.76×10^5^ Vm^−1^), +4.5 kV (3.54×10^5^ Vm^−1^), and +5.5 kV (4.33×10^5^ Vm^−1^). Although Δ*t* was measured at a fixed *z*, a minor correction was made to *z* by considering the ratio of the resultant forward and backward peaks that remained after LASER-2 irradiation.

As expected, (CHCA)H^+^ propagated faster than (CHCA)_2_H^+^; further, the propagation rate increased as the acceleration voltage increased. In classical mechanical treatment, the propagated distance, *z*, is expressed by 
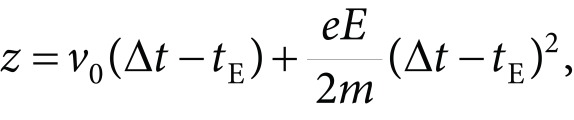
(1) where *t*_E_ is the time required for free molecule ion ejection from surface and/or clusters with mass, *m*, and an initial velocity, *v*_0_, after irradiation by LASER-1, *E* is the electric field for acceleration, and *e* is the elementary charge. As shown by the solid lines in Fig. 5(a), the observed (Δ*t*, *z*) plot of (CHCA)H^+^ is reproduced by Eq. (1) when *t*_E_ is set at 45 ns. The ejection velocity, *v*_0_, was assumed to be 500 ms^−1^, which was obtained from previous reports^15,32)^ although it has been demonstrated that the velocities span a very wide range,^33)^ *v*_0_ was observed to minorly affect propagation because the first term in Eq. (1) was considerably smaller than the second term. Similarly, (CHCA)_2_H^+^ data was reproduced when *t*_E_=65 ns, which was 20 ns behind the ejection of (CHCA)H^+^.

**Figure 6:**
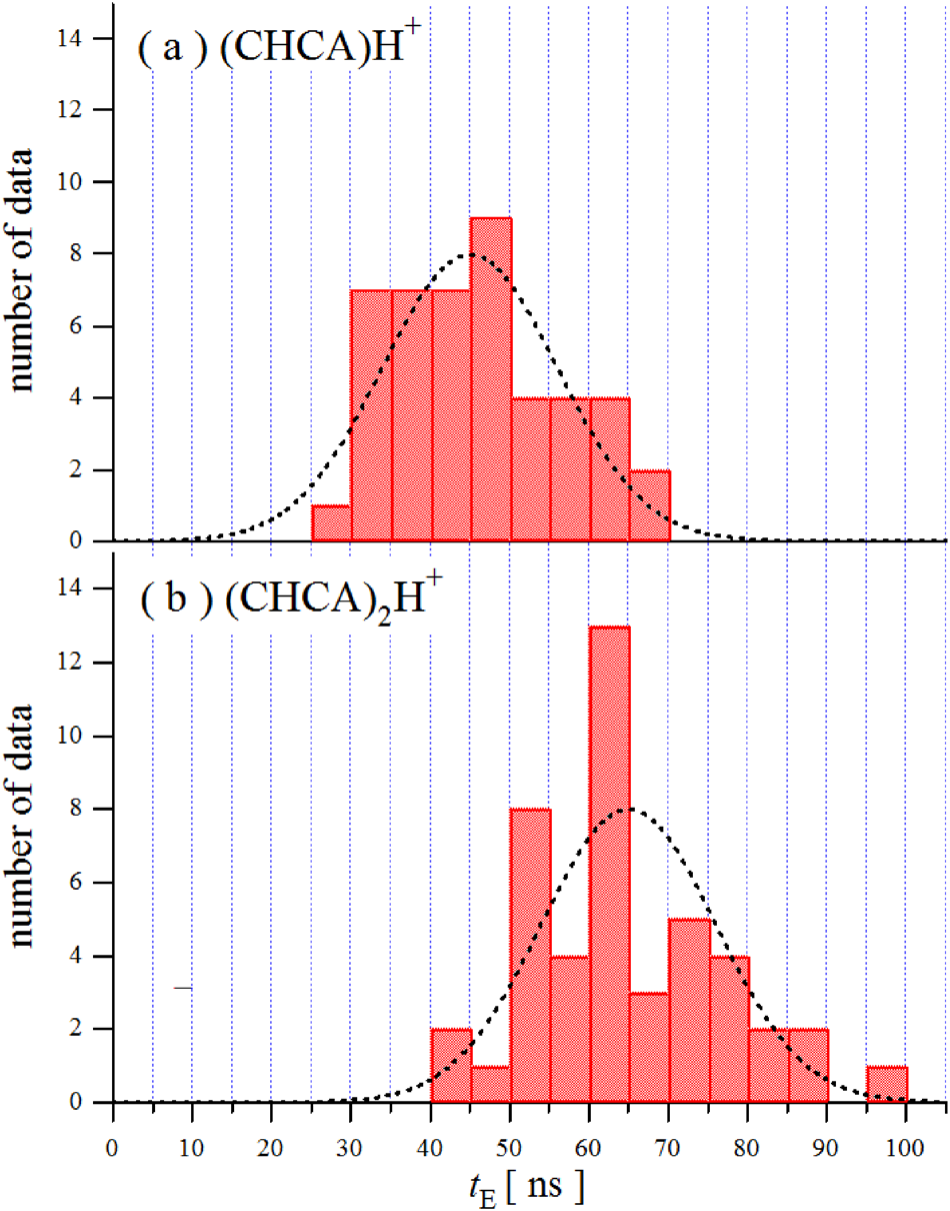
Fig. 6. Distribution of *t*_E_ for (a) CHCAH^+^ and (b) (CHCA)_2_H^+^ obtained from each data in Fig. 5.

Figure 6 shows histograms of the *t*_E_ values determined for each (Δ*t*, *z*) point in Fig. 5. Although the distribution of *t*_E_ exhibited widths as large as 30–35 ns, the difference in *t*_E_ between (CHCA)H^+^ and (CHCA)_2_H^+^ was significant; *t*_E_ of (CHCA)H^+^ was smaller than that of (CHCA)_2_H^+^. The average values of *t*_E_ for (CHCA)H^+^ at three acceleration voltages were 48±10, 49±11, and 40±8 ns at +3.5, +4.5, and +5.5 kV, respectively, whereas those of (CHCA)_2_H^+^ were 70±13, 62±13, and 62±8 ns. The overall average values of *t*_E_ were 46±11 and 64±12 ns for (CHCA)H^+^ and (CHCA)_2_H^+^, respectively. This result indicates that the ejection of (CHCA)_2_H^+^ ion packet into a vacuum occurs approximately 20 ns behind that observed in (CHCA)H^+^ packet ejection. However, it is difficult to judge based on the current data whether the magnitude of the electric field affects the ejection time. Still, it can be said that the electric field does not significantly affect *t*_E_. This result is consistent with the ion formation mechanism proceeding in dense gas and clusters at near the sample surface where collisions dominate.^10,13–24)^

Based on an analysis of Fig. 6, we obtained the following two new observations. (1) The magnitude of the electric field exhibited little effect on the ejection time, indicating that ion ejection was a thermal process and that the released species reflected the composition of the heated condensed phase and/or near the surface of the MALDI target, and (2) it seems that (CHCA)_2_H^+^ was sequentially produced after the formation of (CHCA)H^+^, suggesting that the proton transfer reaction of (CHCA)H^+^+(CHCA)_2_→CHCA+(CHCA)_2_H^+^ represented the main formation process.

## CONCLUSION

We developed a method that can trace the ejected and accelerated MALDI ion packets with a temporal resolution of 10 ns using sheet-like UV laser light. The centers of the packets of (CHCA)H^+^ and (CHCA)_2_H^+^ were observed to eject 46±11 and 64±12 ns after MALDI laser irradiation, respectively. These emission times were not significantly affected by the magnitude of electric field for acceleration, indicating that the emission of ionic species was mainly a thermal process. The delayed ejection of the (CHCA)_2_H^+^ ion packet by approximately 20 ns from the (CHCA)H^+^ ion packet ejection suggested that (CHCA)_2_H^+^ was formed based on a proton transfer reaction from (CHCA)H^+^ to (CHCA)_2_ before vaporization.

The present experimental approach can be applied to various protonated analytes, ions, and neutral species by choosing the laser wavelength, pulse duration, and intensity of LASER-2. Further, we intend to contribute for further understanding the photophysical processes in the MALDI phenomena using this experimental scheme.

## Notes

Mass Spectrom (Tokyo) 2018; 7(1): A0071
